# Tumor Detection and Characterization Using Microwave Imaging Technique—An Experimental Calibration Approach

**DOI:** 10.3390/s26031014

**Published:** 2026-02-04

**Authors:** Anudev Jenardanan Nair, Suraksha Rajagopalan, Naveen Krishnan Radhakrishna Pillai, Massimo Donelli, Sreedevi K. Menon

**Affiliations:** 1 Department of Electrical and Electronics Engineering, Amrita Vishwa Vidyapeetham, Amritapuri 690525, India; anudevj@am.amrita.edu; 2 Centre for Flexible Electronics and Advanced Materials, Amrita Vishwa Vidyapeetham, Amritapuri 690525, India; 3 Department of Electronics and Communication Engineering, Amrita Vishwa Vidyapeetham, Amritapuri 690525, India; 4 Department of Civil, Environmental and Mechanical Engineering, University of Trento, 38123 Trento, Italy; massimo.donelli@unitn.it

**Keywords:** beamforming algorithm, double ridged horn antenna, microwave imaging, relative area of tumor, image reconstruction, SDG3

## Abstract

Microwave imaging (MWI) is a non-invasive technique for visualizing the anomalies of biological tissues. The imaging process is accomplished by comparing the electrical parameters of healthy tissues and malignant tissues. This work introduces a microwave imaging system for tumor detection in breast tissue. The experiment is performed in a homogeneous background medium, where a high dielectric contrast material is used to mimic the tumor. The proposed imaging system is experimentally evaluated for multiple tumor locations and sizes using a horn antenna. Reflection coefficients obtained from the monostatic configuration of the horn antenna are used for image reconstruction. The evaluation metrics, such as localization error, absolute area error, DICE score, Intersection over Union (IoU), precision, accuracy, sensitivity and specificity, are computed from the reconstructed image. A modified version of the beamforming algorithm improves the quality of reconstructed images by providing a minimum accuracy of 96% for all test cases, with an evaluation time of less than 48 s. The proposed methodology shows promising results under a controlled environment and can be implemented for clinical applications after adequate biological studies. This methodology can be used to calibrate any antenna system or phantom, as it has high contrast in conductivity, leading to better imaging. The present study contributes to Sustainable Development Goal (SDG) 3 by ensuring healthy lives and promoting wellbeing for all ages.

## 1. Introduction

As per World Health Organization (WHO) statistics, one in five people suffer from cancer in their lifetime. The mortality rate of this is alarming, with one in nine men and one in twelve women dying from this disease [1]. A study conducted by D. Crosby et al. shows that diagnosed cancers are at an advanced stage in 50% of the cases reported [2]. Early detection of cancer improves the survival rate and quality of life. Traditional imaging systems, such as Computed Tomography (CT scan), Magnetic Resonance Imaging (MRI), Positron Emission Tomography (PET scan), ultrasound and mammography, offer good spatial resolution and sensitivity; however, they have several limitations, including ionizing radiation, high cost, patient discomfort and reduced sensitivity for dense tissues [3].

Microwave imaging (MWI) is a promising alternative to the existing imaging techniques. This technique provides non-ionizing radiation with rapid scanning and can monitor minimal changes in tissue properties [4]. Antennas play a significant role in the MWI system. The performance of the antenna directly influences the image resolution and accuracy of the tumor location. The essential features of an antenna required for microwave imaging include a wider bandwidth, compact size, excellent impedance matching, directional radiation pattern, and low production cost [5].

Microstrip patch, Vivaldi and dielectric resonator antennas are commonly used for microwave imaging. Vivaldi antennas have a compact design and offer a wide bandwidth. Ultra-Wide Band (UWB) antipodal Vivaldi antennas with a 3.6–13 GHz bandwidth and a gain of 9.7 dB were able to detect a 0.9 mm diameter tumor [6]. Another type of Vivaldi antenna working in the frequency band of 3.05–12.2 GHz was reported to detect a 2 mm diameter spherical tumor [7]. A low-profile tapered slot antenna with a bandwidth of 13.87 GHz and a gain of 4.77 dBi was implemented to diagnose breast cancer [8]. This antenna exhibited Specific Absorption Rate (SAR) safety thresholds during breast phantom tests. A side-slotted Vivaldi antenna operating in the band 2.8–7 GHz was used for detecting breast tumors in a heterogeneous phantom model [9]. Beyond the conventional designs, advanced versions of Vivaldi antennas incorporated with metamaterials and array configurations have enhanced the radiation efficiency and tumor detection accuracy [10]. Multistatic microwave imaging with a Vivaldi antenna enables the precise localization of tumors for heterogeneous breast phantoms [11]. A high-gain antenna together with a metasurface neural network classifies tumors into benign and malignant [12]. Conformal 3D-printed hemispherical Vivaldi antenna arrays are used for volumetric imaging and anatomical scenarios [13]. These studies reflect the significance of Vivaldi antennas for high-resolution and clinically viable solutions for microwave imaging.

Patch antennas are also widely used for MWI due to their compact design, ease of fabrication and cost-effectiveness. The adaptability and customization of these antennas make them fit for wearable imaging systems. A UWB patch antenna using a denim substrate, operating from 2 to 11.6 GHz, has been reported for breast cancer imaging [14]. This antenna provides a Voltage Standing Wave Ratio (VSWR) in the range of 1.15 to 2 and can detect tumors as small as 2 mm [15]. Patch antennas have also been used for generating three-dimensional images of breast tumors. A coplanar waveguide (CPW)-fed patch antenna integrated with negative-index metamaterial used for breast cancer imaging has been reported [16]. This antenna exhibits a maximum gain of 7.56 dBi. A metamaterial-based 3D-stacked wideband antenna array was used to classify tumors into malignant and benign. It has an operating range of 1.37–3.16 GHz with a high-fidelity factor [17]. In recent studies, metamaterial and artificial magnetic conductor (AMC) layers on a patch antenna has been implemented to improve gain, directivity and impedance matching. A CPW-fed patch antenna with a metamaterial lens and AMC backing was used for the precise identification of tumors in breast, brain and lung phantom models. This antenna attained a maximum gain of 8.5 dBi at 4.56 GHz [18].

Dielectric Resonator Antennas (DRAs) are also used for microwave imaging applications. These antennas have a compact size, low conduction loss and high radiation efficiency. A UWB DRA with a Sierpinski fractal defected ground structure, operating in the frequency range of 5.6–14.2 GHz with a peak gain of 5.8 dB, offered successful tumor measurement using the principles of the monostatic radar [19]. A compact wideband DRA (6.5–12.5 GHz) was used earlier for the detection of breast cancer [20]. In this antenna, different resonating modes are excited by two asymmetrical dielectric slabs, which enhances the bandwidth.

Horn antennas are among the oldest and most widely utilized in microwave imaging applications due to their high directivity, broad bandwidth and substantial gain. In the domain of MWI, these antennas can be kept as the reference standards for the experimental validation of imaging algorithms and phantom models. A compact 3D-printed double-ridged conical horn antenna detected tumors using both monostatic and bistatic methods, with a specific absorption rate within the explicit limits [21]. A 30% size-reduced version of a standard horn antenna with 1–9 GHz could be used for imaging at 6.2 GHz [22].

The data collected from the antenna have to be reconstructed as images by utilizing different reconstruction algorithms. The Delay and Sum (DAS) algorithm is a widely used imaging technique due to its computational ease and robust performance. The traditional DAS algorithm suffers from high side-lobe levels, noise and limited spatial resolution. To overcome the existing limitations of the DAS algorithm, several modified algorithms are implemented, such as the DAS with Coherence Factor (DAS-CF), *p*th Root Delay and Sum (*p*DAS), Universal Back Projection (UBP), Delay Multiply and Sum (DMAS) algorithm, the combination of the SAR (Specific Absorption Rate) parameter with a modified DAS algorithm, to name a few [23,24,25,26,27]. The Distorted Born Iterative Method (DBIM) is another widely used non-linear image reconstruction algorithm used for solving inverse scattering problems in microwave imaging [28]. In recent years, deep learning and hybrid algorithms have emerged as potential tools for microwave image reconstruction so to overcome the limitations of conventional algorithms. These have transformed conventional reconstruction methods by offering faster processing, improving image quality and reducing sensitivity to noise, making them suitable for clinical applications [29,30,31].

In this study, we have analyzed the performance of the proposed reconstruction algorithm for detecting tumors and quantitatively evaluated various metrics, such as the position, size and shape. For the experimental setup, two tumor-mimicking inclusions (2.25 cm and 1.5 cm in diameter) are individually positioned at nine distinct locations. A standard horn antenna using a monostatic approach is employed for reconstructing tumor-mimicking inclusions from a tissue-mimicking phantom. The changes in the reflection coefficient measured by a horn antenna are used as input for the proposed image reconstruction algorithm. The proposed algorithm is an enhanced version of the conventional DAS-CF beamforming algorithm, integrated with advanced preprocessing, phase correction and coherence factor weighting stages, to suppress the clutter and improve the quality of reconstructed images. The effectiveness of the algorithm is analyzed through various evaluation metrics, including the localization error (LE), absolute area error (AAE), DICE, Intersection over Union (IoU), accuracy, precision, sensitivity, specificity and reconstruction time. The obtained results show improved performance compared to the conventional DAS-CF beamforming algorithm.

## 2. Experimental Analysis for Acquiring Reflection Coefficient

**Figure 1:**
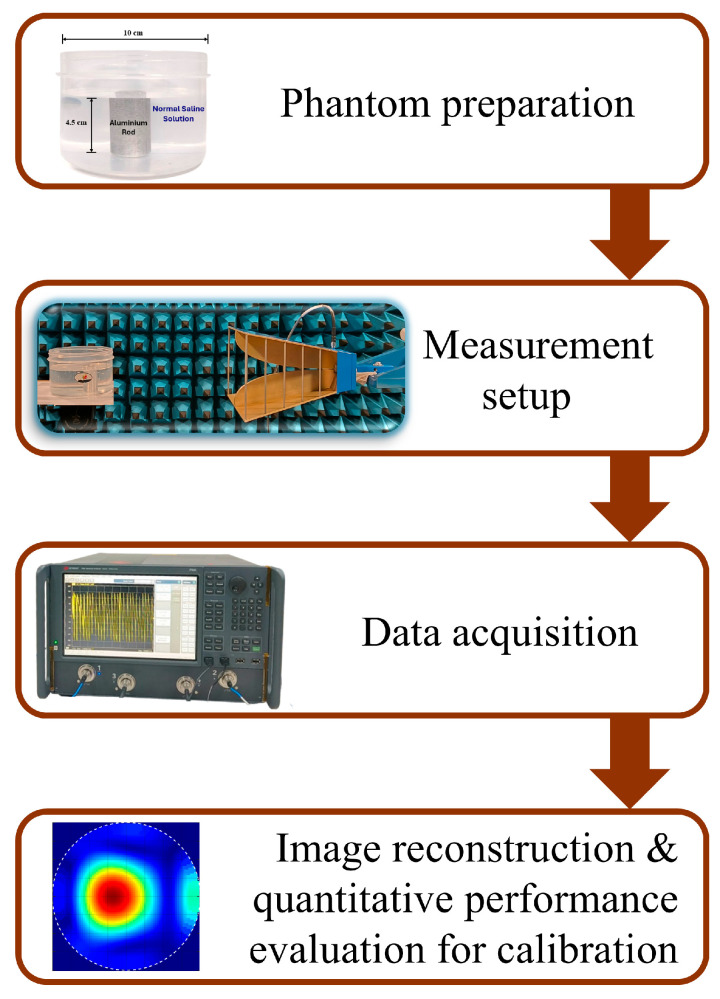
Methodology adopted for the present study.

The methodology adopted for this study is depicted in Figure 1. To acquire the desired data, a tissue-mimicking phantom is developed. The phantom is composed of a plastic cylinder container having a diameter of 10 cm, filled with normal saline (NS) solution (0.9% sodium chloride). An aluminum rod is used to mimic the tumor-like anomalies kept inside the phantom. Two tumor sizes (2.25 cm and 1.5 cm in diameter) are kept at different positions inside the phantom and reflection studies are carried out. This will enable the system to detect and localize the presence of a tumor at various locations.

### 2.1. Phantom Design

The aim of the present work is to evaluate the effectiveness of the propsed image reconstruction algorithm in analyzing the morphological characteristics of tumor-like inclusion using microwave imaging. The proposed phantom consists of normal saline (NS) solution (0.9% NaCl) as background medium and an aluminum rod as inclusion. As per the reported study [32], the dielctric properties of malignant tissue is ten times higher than that of adipose-dominated tissues in the breast. The same property has been implemented in our experimental method to provide a high dielectric contrast with the surrounding medium. An aluminum rod has much higher conductivity than normal saline solution [33,34]. The NS solution is filled in a plastic container with a diameter of 10 cm and a height of 7 cm. The aluminum rod (2.25 cm and 1.5 cm diameter), having height of 4.5 cm, was placed at nine distinct spatial positions across the phantom. This aims to assess the potential of the proposed imaging method to detect, localize and analyze tumor-like anomalies under various scenarios.

**Figure 2:**
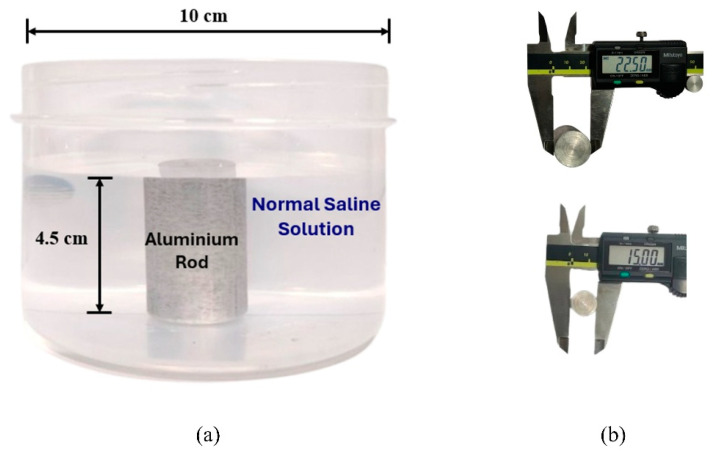
(**a**) Side view of the phantom. (**b**) Measurements of the aluminum inclusions using a digital caliper (all values are in mm).

The phantom model and types of inclusions used for the present study are shown in Figure 2.

### 2.2. Horn Antenna

**Figure 3:**
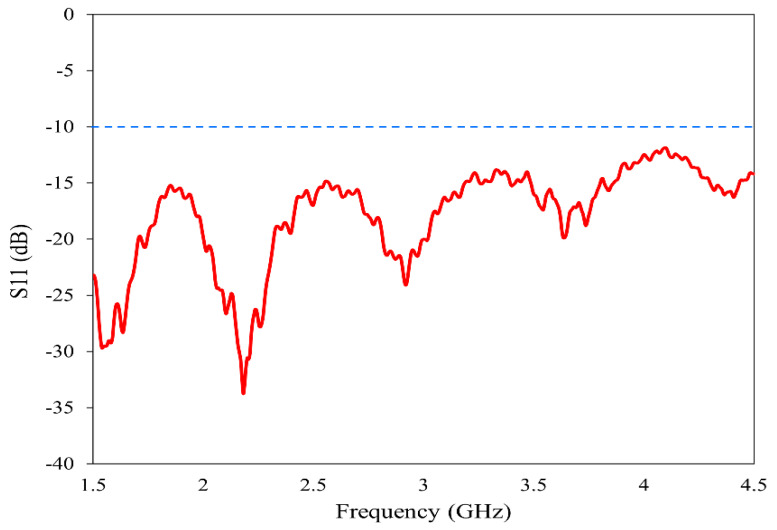
Reflection characteristics of the horn antenna.

The horn antenna (model no: AN 07 047 10 050) is utilized for the proposed imaging. The double-ridged horn antenna has an operating frequency range of 800 MHz to 18 GHz with a gain varying from 4 dBi to 20 dBi. To ensure the suitability of this antenna for our imaging method, we have experimentally analyzed its various characteristics. The reflection coefficient, gain and radiation pattern are measured using the Keysight N5227B Vector Network Analyzer (VNA) (Keysight Technologies, Santa Rosa, CA, USA) in the near-field setup prior to imaging. The operating band is selected from 1.5 GHz to 4.5 GHz to provide a balance between resolution and penetration depth [35,36]. The reflection characteristics of the antenna in the band of study are plotted in Figure 3. In the entire frequency range 2:1 VSWR is maintained, allowing for the data acquisition from the reflection coefficient.

**Figure 4:**
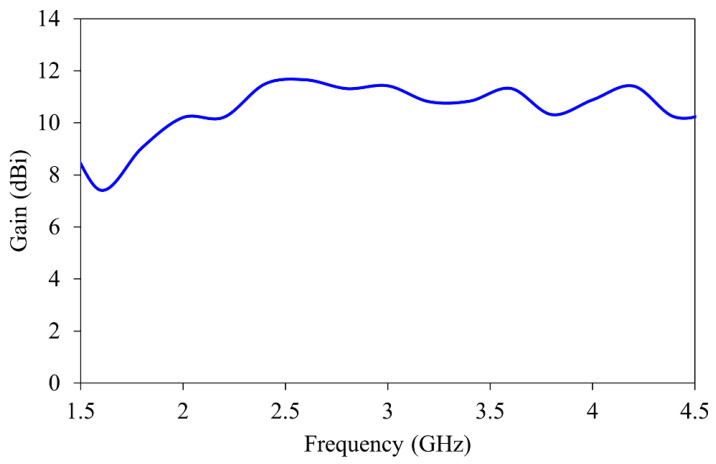
Gain characteristics of the horn antenna.

The gain of the antenna over the specific frequency band is shown in Figure 4. The antenna maintains stable gain between 7.5 and 12 dBi throughout the imaging band. This improves the signal-to-noise ratio during the reconstruction process.

**Figure 5:**
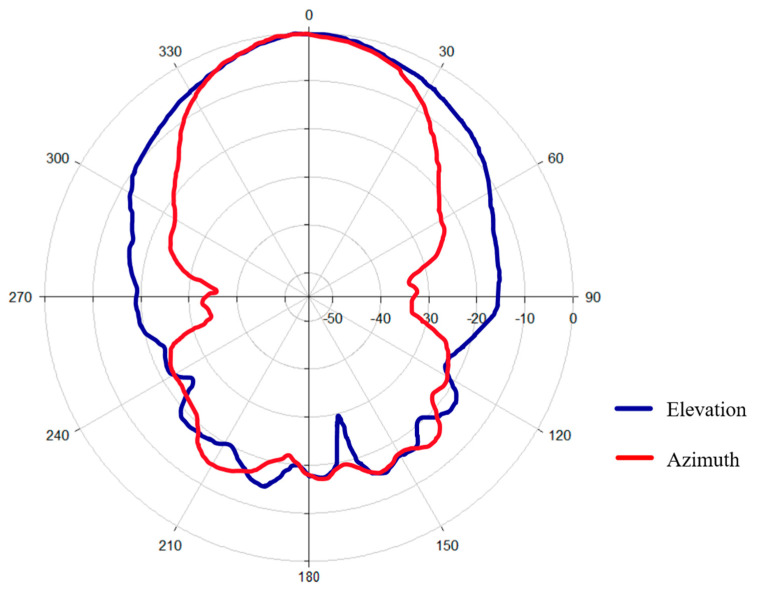
Radiation pattern of the antenna.

The measured radiation pattern (Figure 5) clearly shows that the antenna exhibits a well-defined main lobe in both principal planes. The directional nature of the radiation pattern ensures efficient energy transfer in the target region, with a reduction in unwanted scattering.

### 2.3. Experimental Setup

**Figure 6:**
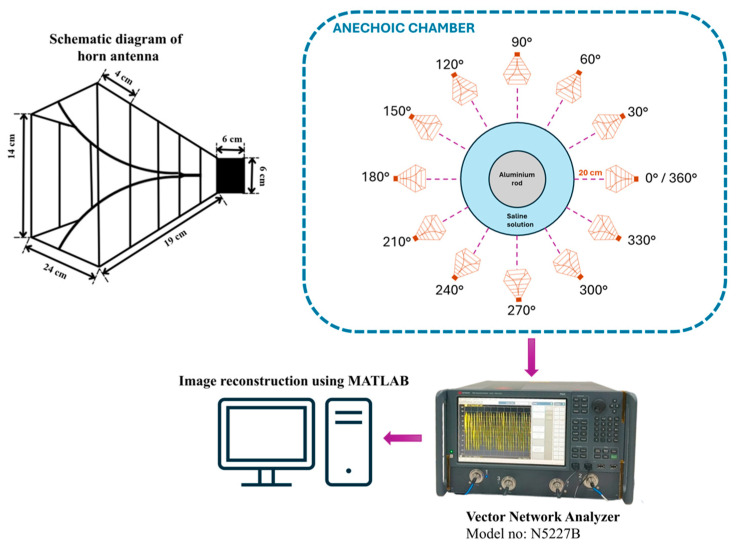
Schematic representation of experimental setup.

**Figure 7:**
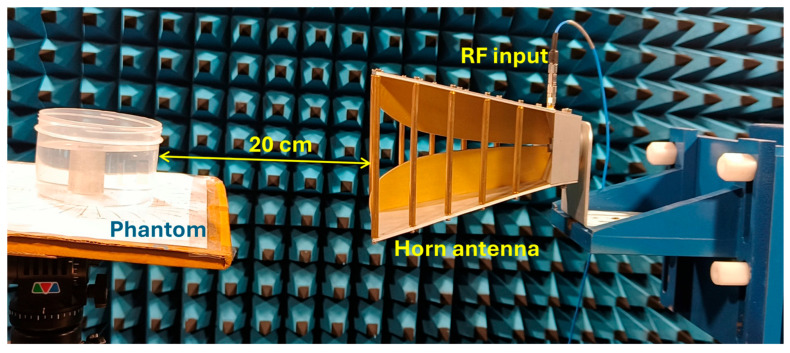
Experimental setup inside the anechoic chamber for measuring reflection coefficients.

The experiment is designed to assess the location and area of the tumor from the designed phantom. Reflection coefficients obtained from the horn antenna are used to specify tumor characteristics with the aid of the image reconstruction algorithm. The antenna configuration follows a monostatic approach, where the antenna remains stationary. The antenna is positioned 20 cm from the phantom, falling within the near-field region. This is desirable to reconstruct images with a high resolution, even for small-sized anomalies. For the present analysis, we use a multi-view acquisition system capable of acquiring data from 12 angular positions with a vector network analyzer (VNA). This is achieved by rotating the phantom in 30° increments. The operating frequency varies from 1.5 GHz to 4.5 GHz, sampled at 502 points. The output power of the VNA (Keysight N5227B) is set to 0 dBm (1 mW). All measurements are conducted in an anechoic chamber to ensure minimum external interference. The schematic representation of the experimental setup for the microwave image reconstruction is shown in Figure 6, where different viewing angles are represented by multiple antenna icons around the phantom. The detailed photograph of actual experimental setup inside the anechoic chamber is shown in Figure 7.

## 3. Image Reconstruction

The proposed algorithm is implemented using MERIT (Microwave Radar-based Imaging Toolbox) for image reconstruction from the reflection data [37]. This algorithm is an enhanced version of DAS beamforming, which will improve the image quality while suppressing noise. The DAS algorithm produces a preliminary image, where the intensity of each pixel is the coherent sum of delayed signals from multiple positions. To enhance image quality, the DAS output is weighted by the coherence factor (CF). This will suppress the incoherent noise or clutter and thereby enhance the target region [38,39,40].

**Figure 8:**
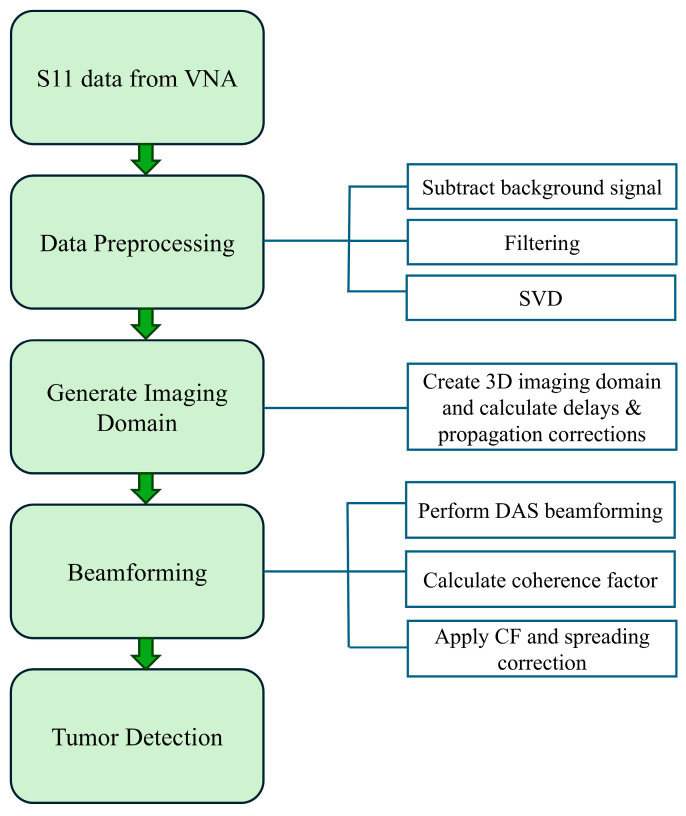
Process flow for MWI reconstruction.

The proposed microwave image reconstruction beamforming method is composed of four main sections: data acquisition, data preprocessing, generation of imaging domain and advanced beamforming for tumor detection and localization. The entire process flow is shown in Figure 8. The initial step for the image reconstruction is the collection of the reflection coefficient from the real-time experiment using a horn antenna in an anechoic chamber. The raw data are collected from horn antenna using a vector network analyzer. In the raw data, each row corresponds to a frequency sample, and each column corresponds to a measurement channel. The measured reflection data contains several unwanted reflections from the antenna and phantom supporting structures, high-frequency noise and powerline harmonics. These raw data undergo various advanced preprocessing stages, including background signal subtraction, filtering and singular value decomposition (SVD). Background signal subtraction is used to minimize static noise, and filtering is used to suppress high-frequency noise. Filtered signals (m×n) are decomposed using the singular value decomposition method to isolate scattered tumor signals from all reflections.

(1) $$S_{filt}=U\Sigma V^{H}$$

where *U* is an m×m unitary matrix and V is an n×n unitary matrix; Σ is an m×n diagonal matrix of singular values (*σ_i_*) arranged in descending order. Large singular values correspond to clutter and smaller ones correspond to scattered response from tumors. After eliminating the first *k* dominant modes, a clean signal was reconstructed from the *N*-*k* components.

(2) $$S_{clean}=\sum_{i=k+1}^{N}\sigma_{i}u_{i}v_{i}^{H}$$

where *N* denotes the total number of singular values determined by the rank of the filtered matrix. The number of modes *k* is selected with respect to the energy-based criterion of the singular values. In the present analysis, singular values accounting for 95% of total energy are removed as clutter and the remaining components are used to reconstruct the image. The choice of this threshold is justified based on improved spatial overlap, reduced localization error, low absolute area error and high specificity across all measurement positions. These steps collectively aim to enhance the signal-to-noise ratio and isolate the scattered signal contributed by the phantom.

Following this step, a three-dimensional imaging domain is created. This represents all focal points where scattering occurs. For our experiment setup, the antenna is stationary and the phantom rotate at an equal increment. This rotation does not affect the beamforming method. The following expressions solely depend on the relative distance between the antenna position and the focal point in the imaging domain.

For each focal point $\vec{r}$, time delay and propagation corrections are calculated.

Time required to travel the signal between *i*^th^ channel and focal point $\vec{r}$:

(3) $$t_{i}\left(\vec{r}\right)=\frac{\left\|\vec{r}-\vec{a_{i}}\right\|\sqrt{\varepsilon_{r}}}{c}$$

where

$\vec{a_{i}}$ is the effective antenna position corresponding to the *i*^th^ channel.

$\varepsilon_{r}$ is the relative permittivity of the medium.

*c* is the speed of light.

The amplitude of the received signal decreases with increasing distance between the antenna and the focal point. This phenomenon is known as spreading loss or propagation attenuation. To compensate for this reduction in signal strength, a propagation (spreading) correction factor ($\lambda_{i}\left(\vec{r}\right))$ is applied during image reconstruction. It is proportional to the square of the distance between the antenna ($\vec{a_{i}}$) and focal point ($\vec{r}$).

(4) $$\lambda_{i}\left(\vec{r}\right)\propto {\left\|\vec{r}-\vec{a_{i}}\right\|}^{2}$$

Let *S_clean_* (*i*,*t*) denote the clutter of the suppressed backscattered signal of *i*^th^ channel obtained after SVD preprocessing. The focused signal contribution from *i*^th^ channel at focal point $\vec{r}$ is defined as

(5) $$c_{i}(t_{i}\left(\vec{r}\right)=\lambda_{i}\left(\vec{r}\right)S_{clean}(i,t_{i}\left(\vec{r}\right))$$

The signal value $S_{clean}(i,t_{i}\left(\vec{r}\right))$ is obtained using interpolation, since the calculated delay $t_{i}\left(\vec{r}\right)$ does not align with discrete sampling instants.

The image intensity (MDAS) at focal point $\vec{r}$ in the imaging domain using DAS beamformer is

(6) $$M_{DAS}\left(\vec{r}\right)=\sum_{i=1}^{N}c_{i}(t_{i}(\vec{r}))$$

The coherence factor at a given point $\vec{r}$ is calculated as

(7) $$CF\left(\vec{r}\right)=\frac{{\left|\sum_{i=1}^{N}c_{i}(t_{i}(\vec{r}))\right|}^{2}}{{N\cdot \sum_{i=1}^{N}\left|c_{i}(t_{i}(\vec{r}))\right|}^{2}}$$

where

$c_{i}$: The back scattered signal from the ith channel, generated from *S_clean_.*

$t_{i}\left(\vec{r}\right)$: The time required to travel between the antenna *i* and focal point $\vec{r}$.

*N*: The total number of channels.

The image intensity after applying coherence factor at each focal point is given as

(8) $$M_{DAS-CF}\left(\vec{r}\right)=CF\left(\vec{r}\right)\;M_{DAS}\left(\vec{r}\right)$$

The final image intensity is calculated after applying spreading correction $\left(\lambda (\vec{r})\right)$ to offset the loss caused by wave propagation.

(9) $$M_{final}\left(\vec{r}\right)=\lambda \left(\vec{r}\right)M_{DAS-CF}\left(\vec{r}\right)$$

The algorithm for microwave imaging using an enhanced version of the DAS-CF algorithm is shown below (Algorithm 1). The detailed pseudocode of the proposed method is provided in the Supplementary Material (Algorithm S1).
**Algorithm 1:** Microwave image reconstruction using enhanced DAS-CF algorithm**Input:** S_11_ data sets (with tumor and without tumor), frequencies, antenna location, imaging domain parameters.**Output:** Reconstructed image, tumor mask, tumor location, tumor area, comprehensive quantitative summary.**Start****Phase I: Data acquisition and preprocessing**Acquire raw reflection data for with tumor (*S*_1_(*f*)) and without tumor (*S*_2_(*f*)) from the VNA.Subtract data without tumor from with tumor to suppress static background:
*S_sub_*(*f*) = *S*_1_(*f*) − *S*_2_(*f*)
Apply frequency domain filtering to reduce high-frequency noise by multiplying a window function *w*(*f*):*S_filt_*(*f*) = *S_sub_*(*f*) *w*(*f*)Apply singular value decomposition (SVD) for removing dominant clutter modes and generating clean signal:$$S_{clean}(f)=\sum_{i=k+1}^{N}\sigma_{i}u_{i}v_{i}^{H}$$**Phase II: Generate imaging domain and delay calculation**Create a 3D imaging domain.For each focal point, calculate the time delay: $$t_{i}\left(\vec{r}\right)=\frac{\left\|\vec{r}-\vec{a_{i}}\right\|\sqrt{\varepsilon_{r}}}{c}$$**Phase III: Beamforming**$\mathrm{Generate}\;\mathrm{time}\;\mathrm{aligned}\;\mathrm{channel}\;\mathrm{signals}:\;c_{i}(t_{i}(\vec{r}))$$\;\mathrm{from}\;S_{clean}(f)$$\mathrm{Compute}\;\mathrm{the}\;\mathrm{image}\;\mathrm{intensity}\;\mathrm{at}\;\mathrm{each}\;\mathrm{focal}\;\mathrm{point}\;\vec{r}$$,\;M_{DAS}\left(\vec{r}\right)$$\mathrm{Compute}\;\mathrm{coherence}\;\mathrm{factor}\;CF\left(\vec{r}\right)$Apply coherence weighting to each focal point:$$M_{DAS-CF}\left(\vec{r}\right)=CF\left(\vec{r}\right)\;M_{DAS}\left(\vec{r}\right)$$$\mathrm{Apply}\;\mathrm{spreading}\;\mathrm{correction}\;(\lambda (\vec{r})$ to compensate for propagation loss:$$M_{final}\left(\vec{r}\right)=\lambda \left(\vec{r}\right)\;M_{DAS-CF}\left(\vec{r}\right)$$**Phase IV: Tumor detection and segmentation**Extract 2D slice at optimum depth.Normalize the image and apply automatic threshold.Perform morphological cleaning.Identify the largest connected region as a tumor mask.Compute centroid of the tumor.Compute tumor area using pixel resolution.**Phase V: Quantitative evaluation**Create ground truth mask.Calculate overlap metrics: DICE, IoU, accuracy, precision, sensitivity and specificity.Calculate localization error and absolute area error.Calculate reconstruction time.**End**

### Reconstruction of Images for Different Inclusions

An automatic thresholding approach has been applied for the image segmentation process to extract the target region from the reconstructed image. To determine the optimal threshold value, a range of threshold values are compared with the DICE score. Maximizing the DICE score ensures that the predicted tumor mask matches the ground truth as closely as possible. The threshold that yields the highest DICE score is selected as the optimal value of segmentation.

**Figure 9:**
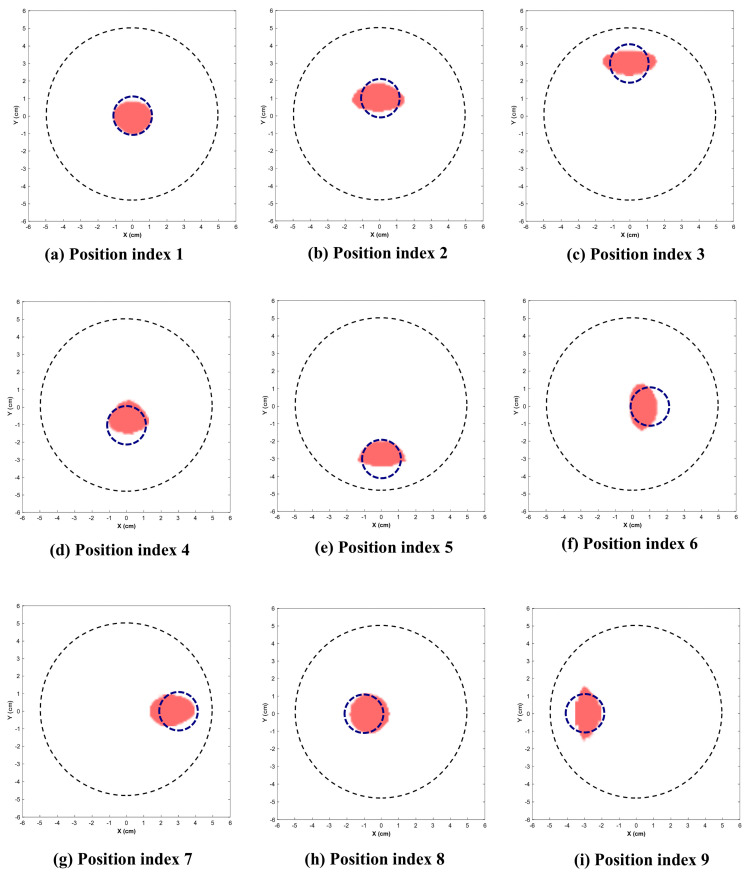
Reconstructed images of 2.25 cm inclusion. (Dotted blue circle represents the actual position and diameter of inclusion.)

**Figure 10:**
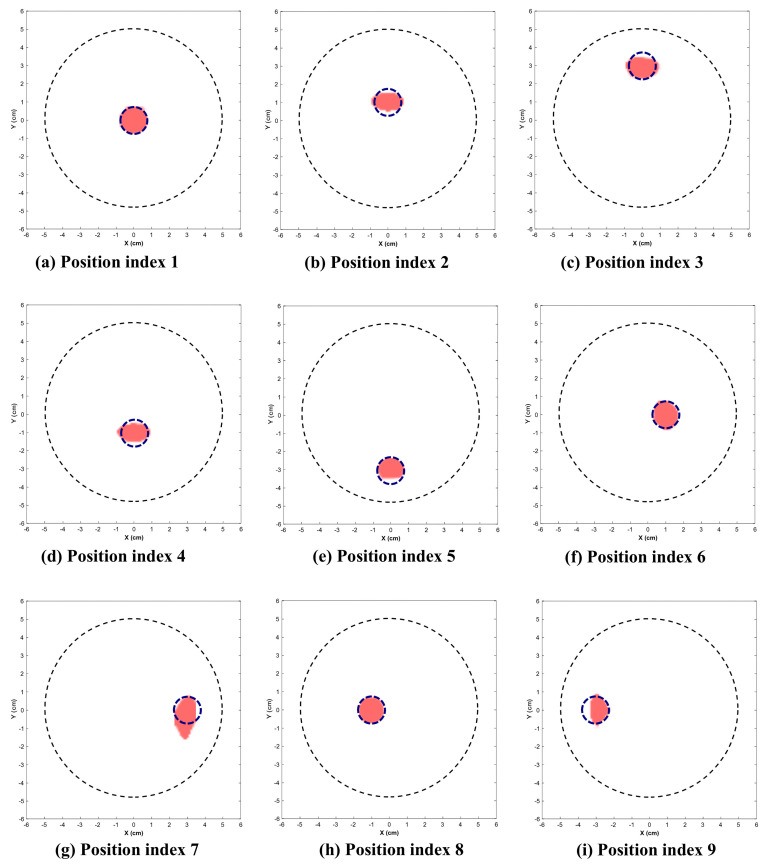
Reconstructed images of 1.5 cm inclusion. (Dotted circle represents the actual position and diameter of inclusion.)

Figure 9 and Figure 10 illustrate the reconstructed images for the 2.25 cm and 1.5 cm diameter inclusion respectively. The dotted circle represents the actual position and diameter of target. Table 1 indicates the position indices where the target is located, along with the reconstructed location and area.

This section presents the visually reconstructed images at each position after segmentation. The following results compare the visual quality of the reconstructed images for two sizes of inclusion at nine positions each. In these images, red is represented by the reconstructed tumor and the dotted blue circle represents the ground truth. The black dotted circle represents the size of the phantom (diameter of 5 cm). The X-axis and Y-axis represent the spatial coordinates (in cm) across the imaging plane of the phantom.

All the images exhibit well-defined target regions at multiple locations. The reconstructed images demonstrate the effectiveness of the proposed algorithm for localizing and delineating various sizes of targets at different locations in the phantom by utilizing the horn antenna. The overall distortion level is minimal due to the calibration of the experimental setup done at the initial stage. This ensures that the reconstructed images are applicable for both quantitative and qualitative analyses.

## 4. Analysis of Reconstructed Image

Analyzing the performance of the MWI system is essential for evaluating the diagnostic precision, effectiveness of the image reconstruction algorithm and clinical viability. To comprehensively assess the performance of the MWI reconstruction system, a range of evaluation metrics has been employed. The localization error (LE), absolute area error (AAE), DICE Similarity Coefficient and Intersection over Union (IoU) are the evaluation metrics considered in this study [41,42]. Diagnostic reliability is further calculated through evaluation metrics such as precision, accuracy, sensitivity and specificity [43]. Reconstruction time is also considered to evaluate the computational efficiency of the proposed method in the calibration experiments. These metrics provide spatial accuracy, similarity in shape, capability for detecting the presence of inclusion and the overall reliability of reconstructed images.

### 4.1. Performance Matrices for Reconstructed Images

#### 4.1.1. Localization Error (LE)

The precise location of the tumor is critical for diagnostic strategy, treatment planning and patient prognosis. The localization error is the Euclidean distance between the actual and reconstructed center coordinates of the inclusion.

(10) $$LE=\sqrt{{\left(x_{actual}-x_{reconst.}\right)}^{2}+{\left(y_{actual}-y_{reconst.}\right)}^{2}}$$

The mean localization error is 0.29 cm for the 2.25 cm inclusion and 0.11 cm for the 1.5 cm inclusion.

#### 4.1.2. Absolute Area Error (AAE)

To assess the shape and size accuracy of the reconstructed image, the absolute area error (AAE) is evaluated for each case under study. The reconstructed area is measured by primarily generating a binary mask from the reconstructed image. The number of foreground pixels within the segmented region is calculated and converted to the reconstructed area.

(11) $$AAE=\left|A_{actual}-A_{reconst.}\right|$$

This metric quantifies the accuracy of the imaging algorithm in terms of identifying the actual and reconstructed area of the inclusion. The mean area error is 0.45 cm^2^ and 0.18 cm^2^ for the 2.25 cm and 1.5 cm inclusions. This indicates that the proposed reconstruction algorithm exhibits reliable performance across multiple positions.

#### 4.1.3. DICE Score and Intersection over Union (IoU)

To quantify morphological similarity between the actual and reconstructed target regions, the DICE score and IoU are computed.

(12) $$DICE\;(R,G)=\frac{2\left|R\cap G\right|}{\left|R\right|+\left|G\right|}$$

where R denotes the set of pixels in the reconstructed area of inclusion and G represents the set of pixels in the ground truth of inclusion.

The IoU measures the overlap between the reconstructed region (X) and the actual region (Y).

(13) $$IoU\;(X,Y)=\frac{\left|X\cap Y\right|}{\left|X\cup Y\right|}$$

The 1.5 cm inclusion achieves a higher DICE score and IoU values across most positions compared to the 2.25 cm inclusion. The average DICE score for the 2.25 cm inclusion is 0.77, and for the 1.5 cm inclusion it is 0.86. The average IoU value is 0.64 and 0.76 for the 2.25 cm and 1.5 cm inclusions respectively.

#### 4.1.4. Pixel-Wise Performance Evaluation

The performance of the reconstructed image is evaluated using four key metrics: precision, accuracy, sensitivity and specificity. For each position, with the help of the predicted tumor position and ground truth mask, pixel-wise comparison was generated. These will provide a comprehensive view of the ability of the system to identify target regions while avoiding false detections. The description and equation of each one is given below.

1. Precision: This is the ratio of the precisely predicted positive cases to the total predicted positive cases. This metric is used to confirm that the pixels used for evaluating the position, area and shape truly belong to the tumor region.

(14) $$Precision=\frac{TP}{TP+FP}$$

2. Accuracy: This indicates the effectiveness of the system to classify the tumor region and healthy region.

(15) $$Accuracy=\frac{TP+TN}{TP+TN+FP+FN}$$

3. Sensitivity: It measures number of true-positive predictions out of all positive predictions. High sensitivity indicates that the proposed algorithm is efficient enough to capture the majority of tumor pixels within the image.

(16) $$Sensitivity=\frac{TP}{TP+FN}$$

4. Specificity: This measures the number of true negatives out of actual negatives. This metric is used to show the effectiveness of the reconstruction algorithm to prevent incorrect classification of healthy tissues as tumors.

(17) $$Specificity=\frac{TN}{TN+FP}$$

(TP—true positive; TN—true negative; FP—false positive; FN—false negative.)

The system demonstrates high accuracy and specificity but at the same time shows variability in precision and sensitivity.

#### 4.1.5. Reconstruction Time

The computational efficiency of the proposed beamforming algorithm is assessed through the reconstruction time. It is recorded in each position for different sizes of targets. All reconstructions are performed on a system with an Intel^®^ Core ™ i5 processor, 16 GB RAM and NVIDIA GeForce RTX3050 GPU running on Windows 11 (64 bit) and MATLAB R2024b. The average reconstruction time for the 2.25 cm and 1.5 cm inclusions is approximately the same (≈42 s). The time variation between each scenario is primarily attributed to the computational cost of the modified DAS-CF beamforming stage. The proposed beamforming stage performs pixel-wise delay calculation and coherent summation for every focal point, every channel and every frequency. The time profiling analysis indicates that the proposed DAS-CF beamforming algorithm accounts for nearly 70–75% of the total running time. The time taken for remaining process stages—data loading, preprocessing, and segmentation—require comparatively negligible time. The difference between in-execution scheduling and memory access patterns during the MATLAB runtime cause minor fluctuations in the reconstruction time. The inclusion size and position do not affect the reconstruction time. These results indicate that the presented imaging system provides acceptable computational performance for calibration purposes.

Table 2 and Table 3 present the performance matrices for the 2.25 cm and 1.5 cm diameter inclusions respectively.

For statistical validation, we have done one-way ANOVA test for the localization error and absolute area error between the two inclusions (2.25 cm and 1.5 cm diameter) across nine distinct positions. The *p*-value for the localization error and absolute area error are 0.019 and 0.0058 respectively. The mean localization error of the 2.25 cm inclusion is 0.293 cm (95% CI: 0.162 cm–0.425 cm), while the 1.5 cm inclusion is 0.112 cm (95% CI: 0.020 cm–0.204 cm). The mean absolute area error is higher (mean: 0.459 cm^2^; 95% CI: 0.281 cm^2^–0.637 cm^2^) than the 1.5 cm inclusion (mean: 0.182 cm^2^; 95% CI: 0.083 cm^2^–0.281 cm^2^). The effect size of the localization error (Cohen’s d ≈ 1.2) and absolute area error (Cohen’s d ≈ 1.5) are large. These results confirm that the size of the tumor affects both the localization error and the absolute area error in a statistically meaningful manner.

To evaluate the effectiveness of the proposed system, a comparison between the average performance of the conventional DAS-CF beamforming algorithm with the modified algorithm is shown in Table 4.

The modified DAS-CF algorithm shows superior performance in comparison with the conventional DAS-CF algorithm. A significant reduction in the localization error (LE) and absolute area error (AAE) is observed in the enhanced version of the DAS-CF algorithm, indicating improved accuracy in both the tumor position and area. The localization error is reduced to 34% and 63% for the 2.25 cm and 1.5 cm diameter inclusions respectively. Similarly, the absolute area error is decreased to 60–63% for both inclusions. DICE and IoU performance metrices quantify the morphological similarity and boundary similarity respectively. In the proposed algorithm, these values are improved (28–58%), indicating an effective overlap between the original region and reconstructed region of the tumor. Precision, accuracy, sensitivity and specificity are the other four metrices used to indicate the quality of the reconstructed image. While maintaining high specificity and accuracy, the proposed method improved the results for precision and sensitivity (11–52%) compared to the conventional algorithm. The reconstruction time is also slightly higher in the proposed system. This is acceptable when considering the image reconstruction quality. The comparison of recent literature studies with the proposed method is presented in Table 5.

The proposed method shows promising results, even though there are some limitations. This study aimed to demonstrate the effectiveness of the enhanced DAS-CF algorithm for image reconstruction of dielectric contrast material (aluminum and normal saline solution). However, real breast tissues are multilayered, and each layer has different dielectric properties, which may influence the effectiveness of the proposed imaging method. Furthermore, the present test setup is not convenient for clinical applications. To address these limitations, future work will focus on developing a patient-friendly wearable antennas and validate its performance using a realistic breast phantom model.

## 5. Conclusions

The present work proposes and validates an experimental calibration methodology for testing the performance of the antenna and reconstruction algorithm for microwave imaging in a controlled environment. The phantom is created using normal saline solution and an aluminum rod in a container to mimic healthy tissue and malignant tissue respectively. The imaging system is evaluated using two separate inclusions with diameters of 2.25 cm and 1.5 cm, each at nine distinct positions. From the reconstructed image, generated from reflection data, we estimated the area and position of the tumor-mimicking inclusion. The evaluation metrics, such as the localization error, absolute area error, DICE score, Intersection over Union (IoU), precision, accuracy, sensitivity and specificity, were also computed. The present analysis has provided a minimum accuracy of 96% for all test cases within a reasonable reconstruction time. This work aligns with Sustainable Development Goal (SDG) 3 by guaranteeing healthy lives and promoting wellbeing for all ages. Future work will focus on developing a wearable antenna with an upgraded reconstruction algorithm implemented on a realistic phantom model.

## Figures and Tables

**Table 1:** Position indices and corresponding coordinates with reconstructed location and area.

Position Index	Coordinates (x, y)	Reconstructed Location (cm)		Reconstructed Area (cm^2^)	
		2.25 cm Dia.	1.5 cm Dia.	2.25 cm Dia.	1.5 cm Dia.
1	(0, 0)	(0, −0.1)	(−0.1, 0)	3.57	1.91
2	(0, 1)	(−0.1, 1)	(0, 1)	3.91	1.59
3	(0, 3)	(−0.1, 2.8)	(0, 2.9)	3.4	1.69
4	(0, −1)	(−0.1, −0.5)	(0, −0.9)	3.41	1.53
5	(0, −3)	(0, −2.8)	(0, −2.9)	3.16	1.48
6	(1, 0)	(0.5, 0)	(1, 0)	3.55	1.79
7	(3, 0)	(2.5, −0.1)	(2.9, −0.4)	4.58	2.12
8	(−1, 0)	(−0.7, 0)	(−0.9, 0)	4.16	1.75
9	(−3, 0)	(−2.8, 0)	(−2.9, 0)	3.48	1.43

**Table 2:** Performance matrices for the 2.25 cm inclusion.

Position Index	LE (cm)	AAE (cm^2^)	DICE	IoU	Precision	Accuracy	Sensitivity	Specificity	Reconstruction Time (s)
1	0.10	0.41	0.94	0.89	0.98	0.99	0.90	0.99	39.40
2	0.10	0.06	0.81	0.68	0.81	0.98	0.81	0.98	38.90
3	0.22	0.57	0.73	0.57	0.78	0.97	0.68	0.98	39.01
4	0.51	0.56	0.70	0.54	0.75	0.97	0.65	0.98	43.44
5	0.20	0.82	0.79	0.66	0.88	0.98	0.72	0.99	42.34
6	0.50	0.43	0.67	0.50	0.70	0.96	0.64	0.98	42.47
7	0.51	0.61	0.75	0.60	0.69	0.97	0.82	0.97	46.84
8	0.30	0.18	0.82	0.69	0.79	0.98	0.84	0.98	42.27
9	0.20	0.49	0.74	0.59	0.78	0.97	0.71	0.98	42.69

**Table 3:** Performance matrices for the 1.5 cm inclusion.

Position Index	LE (cm)	AAE (cm^2^)	DICE	IoU	Precision	Accuracy	Sensitivity	Specificity	Reconstruction Time (s)
1	0.10	0.14	0.95	0.90	0.90	0.99	1.00	0.99	39.89
2	0.00	0.18	0.81	0.68	0.85	0.99	0.78	0.99	41.54
3	0.10	0.08	0.83	0.71	0.84	0.99	0.82	0.99	41.67
4	0.10	0.24	0.82	0.70	0.84	0.99	0.77	0.99	39.78
5	0.10	0.28	0.90	0.82	0.97	0.99	0.84	0.99	47.73
6	0.00	0.02	0.91	0.84	0.90	0.99	0.93	0.99	39.87
7	0.41	0.35	0.71	0.55	0.65	0.98	0.79	0.98	40.62
8	0.10	0.02	0.96	0.91	0.95	0.99	0.96	0.99	40.44
9	0.10	0.33	0.82	0.70	0.91	0.99	0.75	0.99	41.16

**Table 4:** Average performance comparison between the proposed imaging system with the conventional DAS-CF algorithm.

Parameter	2.25 cm Diameter Inclusion			1.5 cm Diameter Inclusion		
	Conventional	Modified	Improvement	Conventional	Modified	Improvement
Localization Error (LE) (in cm)	0.44	0.29 ↓	34.1%	0.3	0.11 ↓	63.3%
Absolute Area Error (AAE) (in cm^2^)	1.16	0.46 ↓	60.3%	0.49	0.18 ↓	63.3%
DICE	0.6	0.77 ↑	28.3%	0.65	0.86 ↑	32.3%
IoU	0.46	0.64 ↑	39.1%	0.48	0.76 ↑	58.3%
Precision	0.62	0.8 ↑	29%	0.78	0.87 ↑	11.5%
Accuracy	0.97	0.97	No change	0.99	0.99	No change
Sensitivity	0.67	0.75 ↑	11.9%	0.56	0.85 ↑	51.8%
Specificity	0.98	0.98	No change	0.99	0.99	No change
Reconstruction time (s)	39	42 ↑	7.8% (slower)	38.7	41.4 ↑	7% (slower)

**Table 5:** Comparison of the proposed imaging system with the state of the art.

References	Type of Antenna	Operating Frequency Range (GHz)	Reconstruction Algorithm	Evaluation Method	Target Pattern	Imaging Outcome & Validation
[6]	Antipodal Vivaldi antenna	3.6–13 GHz	DMAS	Monostatic	Single target, single location	Target localization (qualitative validation)
[7]	Antipodal Vivaldi antenna	3.05–12.2	DMAS	Monostatic	Single target, single location	Target localization (qualitative validation)
[9]	Side slotted Vivaldi antenna	2.8–7	Iteratively Corrected Delay and Sum (IC-DAS)	Multistatic	Single and multiple targets and different spatial positions	Target localization; validation based on signal-to-mean ratio (SMR) and visual inspection
[11]	Vivaldi	1.1–10	DAS with new preprocessing step	Multistatic	Multiple targets, multiple locations	Target localization; validation based on signal-to-mean ratio (SMR) and visual inspection
[15]	UWB patch antenna	3.1–11.6	Microwave Confocal Imaging (MCI) with IFFT+ calibration + clutter removal + synthetic focusing	Multistatic	Single target, single location	Target localization (qualitative validation)
[16]	Patch antenna	2.42–3.3 and 4–15	DAS and DMAS	Multistatic	Single target, single location	Target localization (qualitative validation)
[17]	3D stacked antenna	1.37–3.16	Iteratively Corrected Coherence Factor Delay-Multiply-and-Sum (IC-CF-DMAS)	Multistatic	Multiple size targets, multiple locations	Target localization; qualitative validation; identified benign and malignant tumors
[19]	Fractal DRA	5.6–14.2	DAS-CF and DMAS-CF	Monostatic	Single target, single location	Target localization and size estimation (qualitative validation)
[20]	DRA	6.5–12.5	Mean value-based imaging + GPR (Ground Penetrating Radar) algorithm	Monostatic	Single target, single location	Target localization and depth estimation (qualitative validation)
[21]	Horn antenna	0.69–12	Fundamental DAS+ Weighting approach+ Filtering	Monostatic and bistatic approach	Multiple targets with same size and multiple positions	Target localization (qualitative validation)
[27]	Vivaldi antenna	-	Improved DAS algorithm	Monostatic	Multiple size targets, single position	Target localization (qualitative validation)
Present study	Horn antenna	1.5–4.5	Modified version of DAS-CF algorithm	Monostatic	Multiple size targets, multiple positions	Target localization and area estimation with quantitative validation using DICE score, IoU, precision, accuracy, sensitivity and specificity

## Data Availability

Data available on request.

## Supplementary Materials

The following supporting information can be downloaded at: https://www.mdpi.com/article/10.3390/s26031014/s1, Algorithm S1: Enhanced DAS–CF Microwave Image Reconstruction.

## Abbreviations

The following abbreviations are used in this manuscript:

SDG	Sustainable Development Goal
IoU	Intersection over Union
WHO	World Health Organization
MRI	Magnetic Resonance Imaging
PET	Positron Emission Tomography
MWI	Microwave Imaging
UWB	Ultra-Wide Band
SAR	Specific Absorption Rate
VSWR	Voltage Standing Wave Ratio
AMC	Artificial Magnetic Conductor
DRA	Dielectric Resonator Antenna
DAS	Delay and Sum
DAS-CF	DAS with Coherence Factor
pDAS	pth Root Delay and Sum
UBP	Universal Back Projection
DMAS	Delay Multiply and Sum
DBIM	Distorted Born Iterative Method
NS	Normal Saline
VNA	Vector Network Analyzer
SVD	Singular Value Decomposition
MERIT	Microwave Radar-based Imaging Toolbox
LE	Localization error
AAE	Absolute Area Error
