# Supplementary material for: Tumor Detection and Characterization Using Microwave Imaging Technique—An Experimental Calibration Approach
Source: Sensors (Basel). 2026 Feb 4;26(3):1014. doi: 10.3390/s26031014 (PMC12900151; doi:10.3390/s26031014)
Supplement: Supplementary file 1 [file sensors-26-01014-s001.zip › sensors-4100159-supplementary.pdf]

```
Algorithm 1: Enhanced DAS–CF Microwave Image Reconstruction
input:
        S_target ∈ ℂ^{K×N}             ▹ S11 data with tumor
        S_reference ∈ ℂ^{K×N}          ▹ S11 data without tumor
        f[1:K]                         ▹ frequency vector
        a[1:N] ∈ ℝ³                    ▹ antenna coordinates
        ε_r                            ▹ relative permittivity
        r_domain ∈ ℝ³                  ▹ 3-D imaging domain points
        τ_th                           ▹ segmentation threshold
output:
        TumorMask, TumorCentroid, TumorArea, Metrics

────────────────────────────────────────────────────────────
Phase I — Data Preprocessing
────────────────────────────────────────────────────────────
signals ← S_target − S_reference                 ▹ background removal
Apply GaussianWindow(signals)                    ▹ suppress HF noise
signals_clean ← SVD_RemoveClutter(signals)       ▹ clutter reduction

────────────────────────────────────────────────────────────
Phase II — Delay Computation & Imaging Volume
────────────────────────────────────────────────────────────
[points, axes] ← ImagingDomain(radius, resolution)
for each point r ∈ points do
    for each antenna i = 1…N do
        d_i ← || r − a[i] ||                     ▹ propagation distance
        τ_i(r) ← d_i √ε_r / c                    ▹ propagation delay
        S_delayed[i] ← signals_clean[i] · exp(−j2πfτ_i(r))
    end for
end for

────────────────────────────────────────────────────────────
Phase III — DAS Beamforming with CF Weighting
────────────────────────────────────────────────────────────
for each point r ∈ points do
    M_DAS(r) ← Σ_i S_delayed[i]                  ▹ classical DAS
    CF(r) ← |Σ_i S_delayed[i]|² / (N Σ_i |S_delayed[i]|²)
    M_CF(r) ← M_DAS(r) · CF(r)                   ▹ coherence weighting
    λ(r) ← SpreadingCorrection(r)
    M_final(r) ← λ(r) · M_CF(r)                  ▹ corrected intensity
end for

────────────────────────────────────────────────────────────
Phase IV — Tumor Segmentation
────────────────────────────────────────────────────────────
slice ← ExtractSlice(M_final, z_opt)
slice_norm ← Normalise(slice)
mask_bin ← (slice_norm > τ_th)                   ▹ thresholding
mask_clean ← MorphClean(mask_bin)                ▹ remove noise
TumorMask ← LargestConnectedRegion(mask_clean)
```

```
TumorCentroid ← ComputeCentroid(TumorMask)
TumorArea ← CalculateArea(TumorMask)

────────────────────────────────────────────────────────────
Phase V — Quantitative Evaluation
────────────────────────────────────────────────────────────
GT_mask ← CreateGroundTruth()
Metrics ← ComputeMetrics(TumorMask, GT_mask)     ▹ Dice, IoU, etc.
return TumorMask, TumorCentroid, TumorArea, Metrics

End Algorithm
```
